# Cost‐effectiveness of e‐cigarettes compared with nicotine replacement therapy in stop smoking services in England (TEC study): a randomized controlled trial

**DOI:** 10.1111/add.14829

**Published:** 2019-12-04

**Authors:** Jinshuo Li, Peter Hajek, Francesca Pesola, Qi Wu, Anna Phillips‐Waller, Dunja Przulj, Katie Myers Smith, Natalie Bisal, Peter Sasieni, Lynne Dawkins, Louise Ross, Maciej Lukasz Goniewicz, Hayden McRobbie, Steve Parrott

**Affiliations:** ^1^ Mental Health and Addiction Research Group, Department of Health Sciences University of York York UK; ^2^ Queen Mary University of London London UK; ^3^ King's College London London UK; ^4^ London South Bank University London UK; ^5^ Leicester City Council Leicester UK; ^6^ Roswell Park Comprehensive Cancer Centre Buffalo NY USA

**Keywords:** Cost‐effectiveness, e‐cigarette, economic evaluation, life‐time modelling, Markov model, nicotine replacement therapy, smoking cessation, stop smoking services

## Abstract

**Aim:**

To evaluate the cost‐effectiveness of e‐cigarettes as a smoking cessation aid used in routine stop smoking services in England.

**Design:**

Cost‐effectiveness analysis was performed from the National Health Service (NHS) and Personal Social Services (PSS) perspective for 12‐month periods and life‐time. Costs, including that of both treatments, other smoking cessation help and health‐care services, and health benefits, estimated from EQ‐5D‐5L and measured in quality‐adjusted life‐years (QALYs), for the 12‐month analysis, came from a randomized controlled trial. Life‐time analysis was model‐based with input from both trial data and published secondary data sources. Cost‐effectiveness was measured by an incremental cost‐effectiveness ratio (ICER).

**Setting:**

Three stop‐smoking service sites in England.

**Participants:**

Adult smokers (*n* = 886) who sought help to quit in the participating sites.

**Intervention and comparator:**

An e‐cigarette (EC) starter kit versus provision of nicotine replacement therapy (NRT) for up to 3 months, both with standard behavioural support. A total of 886 participants were randomized (439 in the EC arm, 447 in the NRT arm). Excluding one death in each arm, the 1‐year quit rate was 18.0 and 9.9%, respectively.

**Measurements:**

Cost of treatments was estimated from the treatment log. Costs of other smoking cessation help and health‐care services and EQ‐5D‐5 L were collected at baseline, 6‐ and 12‐month follow‐ups. Incremental costs and incremental QALYs were estimated using regression adjusting for baseline covariates and their respective baseline values.

**Findings:**

The ICER was £1100 per QALY gained at the 12 months after quit date (87% probability below £20 000/QALY). Markov model estimated the life‐time ICER of EC to be £65 per QALY (85% probability below £20 000/QALY).

**Conclusion:**

Using e‐cigarettes as a smoking cessation aid with standard behavioural support in stop‐smoking services in England is likely to be more cost‐effective than using nicotine replacement therapy in the same setting.

## Introduction

In Great Britain, the prevalence of e‐cigarette (EC) use in adults in 2017 was approximately 6% of the adult population 1. The policy on EC varies internationally, and whether or not it should be promoted as a way to quit smoking remains a controversial issue 2.

The UK National Institute for Health and Care Excellence (NICE) guidance for Stop‐Smoking Service (SSS) currently advises that ‘people who smoke should not be discouraged from switching to e‐cigarettes, and as a result continue to smoke’ 3. The evidence base is still developing, and further research on effectiveness and cost‐effectiveness of EC is needed to inform policy.

We conducted a two‐group, pragmatic, multi‐centre, individually randomized controlled trial (RCT) comparing EC with nicotine replacement therapy (NRT) within the English SSS (National Research Ethics Service approval 14/LO/2235). The protocol has been published previously 4 and the carbon monoxide (CO)‐validated 12‐month sustained abstinence rate was 9.9% [standard error (SE) = 1.4%] in the NRT arm and 18.0% (SE = 1.7%) in the EC arm 5. The project has been published in full in Health Technology Assessment 6. In this article we present the analyses to: (1) evaluate 12‐month cost‐effectiveness of EC comparing with NRT from a National Health Service (NHS) and Personal Social Services (SSS) perspective; (2) observe if the participants spend more on smoking cessation due to EC; and (3) estimate life‐time cost‐effectiveness of EC comparing with NRT from a NHS/PSS perspective.

## Methods

### Trial design

#### Intervention and comparator

All participants were offered six weekly behavioural support sessions at their SSS as per standard practice, with the second session on the target quit date (TQD).

Participants in the NRT arm (the comparator) could choose two products and were free to switch products. The trial sites provided NRT products either directly or through a letter of recommendation (LOR) to use in local pharmacies (for details see Supporting information). Direct provision was free of charge, while LOR imposed a prescription charge upon redeeming if not exempted. Supplies were provided for up to 3 months, as per usual practice, and could be obtained subsequently through GP prescription.

The EC arm, the intervention, was provided with the ‘One Kit’ device and a 30‐ml bottle of e‐liquid (18 mg/ml nicotine). Due to the discontinuation of the original product, the One Kit 2016 was given to a small group of participants entering the trial at a later time (for device details, see Supporting information). Participants were instructed to obtain further e‐liquid supplies themselves and advised on possible channels of purchase. Information sheets on how to operate the EC were also provided. One additional 10‐ml bottle of e‐liquid could be requested if required.

The initiation of NRT or EC use started immediately after randomization on TQD.

#### Participants

Participants were recruited from three SSS sites in England. Smokers aged 18 years or over, who sought help to quit and were able to read, write and understand English, were eligible for the trial. Those who were pregnant or breastfeeding, had a strong preference to use or not to use NRT or EC in their quit attempt or were currently enrolled in other interventional research or currently using NRT or EC were excluded. Written informed consent was obtained at baseline.

From May 2015 to January 2017, 886 participants were randomized (447 in the NRT arm and 439 in the EC arm). The median age was 41 [interquartile range (IQR) = 33–51] in the NRT arm and 41 (IQR = 33–53) in the EC arm. Males represented 52% (228 of 439) of the EC arm and 52% (233 of 447) of the NRT arm. One death occurred before 6‐month follow‐up in the NRT arm and one death occurred before 12‐month follow‐up in the EC arm.

#### Blinding

It was not possible to mask the allocation when conducting the cost‐effectiveness analysis. However, the data were not accessed by the health economists before data lock, and the smoking cessation outcome data were only made available as an input to the model‐based secondary analysis after the primary analysis was completed.

### Data collection

#### Costs

All costs and expenses are presented in 2015/16 pounds sterling (£). The Supporting information, Table S1 shows all the unit costs used in the analysis.

##### Treatment cost

Treatment costs consisted of training and delivery costs. Training for SSS advisers on EC use was a 1‐hour session delivered once at each site by two members of the research team. A total of 30 advisers attended the training. Each adviser was equipped with one demonstration One Kit at a cost of £19.35 per kit, including liquid and accessories. The advisers were costed at mid‐point of the NHS pay bands 5 and 6. The two trainers were costed at the NHS pay band 6. Including salary on‐costs, overheads and capital, the cost was estimated at £37 per hour for advisers and £42 per hour for trainers 7. We assumed that all advisers had received routine training in behavioural support and NRT use on the job, so these costs only applied to the EC arm and the NRT arm did not require extra training.

For treatment delivery, attendance of weekly support sessions and the provision of NRT, LORs or EC were recorded at each session. We assumed that all LORs issued would be redeemed, and therefore the cost of prescribed NRT products also incurred. NRTs were costed at their weighted average net ingredient cost (NIC) per prescription item by form and dosage plus dispense fee 8, 9. The cost of EC and e‐liquid provided by the study and the printing cost of EC leaflets and pharmacy lists were recorded by the research team. Only the sessions attended and EC or NRT issued on record were costed.

##### Smoking cessation help costs and health‐care services costs outside the trial

Smoking cessation and other health‐care services utilization and quantities outside the trial were collected through self‐reported questionnaire at baseline, 6‐ and 12‐month follow‐up for the previous 6‐month period. Quantities were then multiplied by the unit costs of the services or weighted average NIC plus the dispensing fee of prescribed items using secondary data sources 7, 8, 9, 10, 11, 12, 13.

##### Participants’ expenses on smoking cessation

EC purchasing expenses (including refills), NRT over‐the‐counter and prescription charges were estimated in both arms based on self‐reported data collected at baseline, 6‐ and 12‐month follow‐ups. NRT over‐the‐counter expenses were estimated using the quantities of the products multiplied by the NIC plus dispensing fee 8, 9. EC expenses were reported in monetary terms. The prescription charges were costed at £8.2 per item where applicable 14.

#### Quality‐adjusted life‐years (QALYs)

The 5‐level EuroQol 5‐dimension (EQ‐5D‐5L) questionnaire was used to measure health‐related quality of life at baseline, 6 and 12 months 15. It consists of five domains (mobility, self‐care, usual activities, pain/discomfort, anxiety/depression), each with five levels of severity ranging from no problem to severe problem, and a visual analogue scale (EQ VAS) ranging from 0 to 100, with a higher score reflecting better health on the day. Following the NICE statement on valuation set at the time of the analysis, the recommended mapping function was used to calculate utility values 16, 17. QALYs were then derived by calculating the area under the curve from baseline to 6 months and 6–12 months 18.

### Missing data

Missing data at baseline and follow‐ups were handled by multiple imputation following Rubin's rules, assuming missing‐at‐random 19. The imputation was performed by treatment arms. The imputation model included the following variables: training cost, intervention delivery costs, smoking cessation help costs, pharmacotherapy costs, health‐care services use costs and EQ‐5D (VAS and utility values) at baseline, 6‐ and 12‐month follow‐ups, age, gender, ethnicity, study site, Fagerström Test of Cigarette Dependence (FTCD) at baseline, entitlement of free prescriptions, expenses on NRT over‐the‐counter, EC purchase and prescription charges. A chained equation model was developed and predictive mean matching was used as the imputation method, using the 10 nearest neighbours to the prediction as a set to draw from. As a rule of thumb, the number of imputations was set to approximately the highest percentage of missing data in all variables 20. Costs and QALYs information for those patients who died were replaced with zero after the date of death.

As smoking cessation outcomes were not revealed to health economists before completion of the primary analysis, the cessation rate at 12 months after quit date was not imputed with other variables. Those who were lost to follow‐up or had no CO reading were classified as smoking, and those who had died were excluded from the calculation.

### Primary analysis

The analysis was undertaken according to a pre‐specified analysis plan 21. The primary analysis was an incremental cost‐effectiveness analysis on an intention‐to‐treat basis from an NHS and PSS perspective during the 12‐month trial period 22. The total costs consisted of treatment cost and the costs to the SSS and NHS (smoking cessation services cost outside the trial and health‐care services use costs) during the 12‐month period. The difference in costs between arms was estimated by a generalized linear regression model controlling for the costs to the SSS and NHS at baseline, age, gender, study site, entitlement of free prescriptions and FTCD at baseline. The effectiveness was presented in terms of QALYs, the difference in which was estimated by a generalized linear regression model controlling for utility value at baseline, age, gender, study site, entitlement of free prescriptions and FTCD at baseline. By dividing the difference in total costs by the difference in QALYs, an incremental cost‐effectiveness ratio (ICER) was calculated to measure the additional cost per QALY gained by EC, compared with NRT. It was then measured against the NICE recommended willingness‐to‐pay (WTP) threshold of £20 000 and £30 000 per QALY gained 22. Neither costs nor QALYs were discounted, as they were collected within 1 year.

Uncertainty surrounding the ICER was assessed through a non‐parametric bootstrap re‐sampling technique 23. Bootstrap randomly drew individuals from the original sample by arm to construct a slightly different replicate sample with the same sample size. Each bootstrap iteration then estimated the incremental costs and QALYs based on the replicate sample of that iteration. A cost‐effectiveness plane (CEP) and cost‐effectiveness acceptability curves (CEACs) were plotted with 5000 bootstrapped estimates 24.

### Secondary analyses

To assess the impact of imputation, a complete case analysis (CCA) was undertaken using the same regression method in the primary analysis. Only the participants who had complete data on all variables in the regression model were included.

Currently, participants carry the whole financial burden of EC after the initial pack, while NRT could be acquired on prescription for a longer period. To assess if provision of a free starter kit for smoking cessation shifts the later cost burden to smokers, participants’ expenses on smoking cessation aids were estimated and compared descriptively between arms.

**Figure 1:**
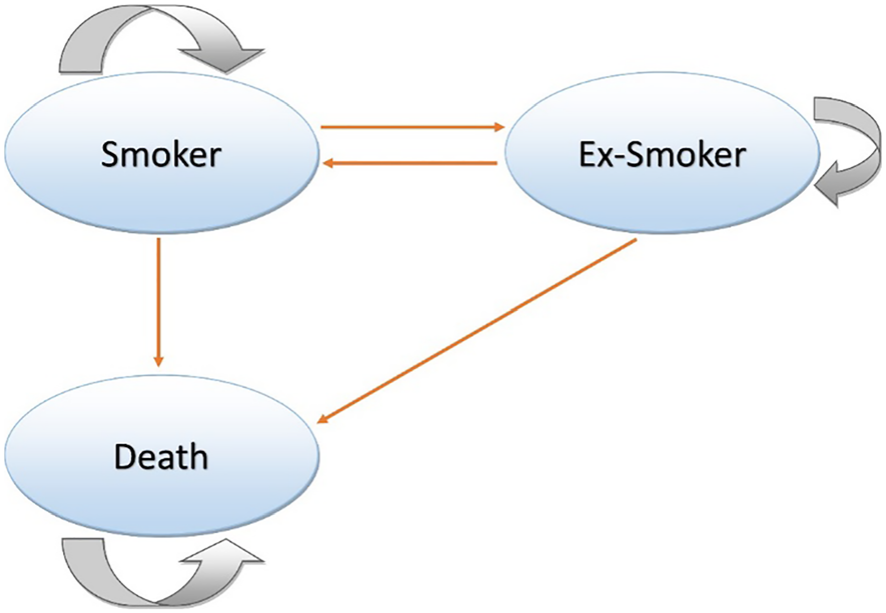
Schematic representation of Markov model [Colour figure can be viewed at wileyonlinelibrary.com]

A Markov model used in a previous trial was updated and used to project long‐term costs and effectiveness 11. Figure 1 illustrates the three‐state model structure: smoker, ex‐smoker and death. The arrows between states indicate the possible pathways of transition and their direction. The model simulated a cohort of 1000 smokers who were assigned to the states proportionally, according to the 1‐year quit rate from trial results at the end of the first cycle of the model. An annual relapse rate of 10% was applied for the following 10 years and abstinence was subsequently assumed to be permanent 25, 26, 27.

Deaths that occurred during the trial were used to estimate the mortality rate of the first cycle. The long‐term mortality rates were obtained from the deaths registered in England and Wales in 2016 by the Office for National Statistics, adjusted for the increased risk for smokers and ex‐smokers based on the Doll's British doctors’ study 28, 29. The model then ran on 1‐year cycles until the survivors reached their 90th year, which could be considered a life‐time horizon. It was assumed that no attempt to quit was made during the modelling period.

The total costs and QALYs estimated from the trial were taken as costs and effectiveness input for the first cycle of the model. Table 1 shows the model inputs after the first cycle estimated from various studies 25, 29, 30, 31, 32. The model took into account the life‐time incidence of smoking‐related diseases and the costs of secondary care for treating smoking‐related diseases. Patients’ utilization of hospital in‐patient care was derived from Hospital Episode Statistics 33 and combined with the NHS reference costs 12 to calculate the annual costs of secondary care for smoking‐related diseases by age and gender. Using the methods introduced by the World Health Organization Economics of Tobacco Toolkit 34, the costs attributable to smoking for smokers and ex‐smokers were estimated by multiplying the calculated annual costs for smoking‐related diseases by their respective smoking‐attributable proportion (Eqn (1)). These attributable costs formed the long‐term cost inputs after the first cycle of the model.

(1) $$\text{Smoking attributable proportion}=\frac{p_{\mathrm{cur}}\left(r_{\mathrm{cur}}-1\right)+p_{\mathrm{ex}}\left(r_{\mathrm{ex}}-1\right)}{1+p_{\mathrm{cur}}\left(r_{\mathrm{cur}}-1\right)+p_{\mathrm{ex}}\left(r_{\mathrm{ex}}-1\right)}$$

Where:

- p_cur_/ p_ex_ = smoking prevalence/proportion of ex‐smokers; and
- r_cur_/r_ex_ = increased risk for having smoking‐related diseases for current/ex‐smokers compared to people who never smoked.

The annual QALYs were derived from the EQ‐5D utility values based on a study of Health Survey for England data, with a sample size of 13 241 30. Both costs and QALYs were discounted at a yearly 3.5% rate beyond 12 months after randomization 22.

**Table 1:** Model inputs from the literature.

Parameters	Value (SE)		Source
Annual probability of relapse			
In the 10 years after first cycle	10.00% (3.06%)		25, 26, 27
After 10 years after first cycle	0%		25, 26, 27
Mortality			
Male age group (years)	Continuing smokers	Ex‐smokers	
35–44	0.24% (0.40%)	0.18% (0.35%)	28, 29
45–54	0.80% (0.40%)	0.51% (0.73%)	28, 29
55–64	1.94% (0.52%)	1.24% (0.58%)	28, 29
65–74	5.15% (0.82%)	3.08% (0.59%)	28, 29
75+	25.36% (2.04%)	15.12% (1.14%)	28, 29
Female age group (years)	Continuing smokers	Ex‐smokers	
35–44	0.14% (0.31%)	0.11% (0.27%)	28, 29
45–54	0.53% (0.33%)	0.34% (0.59%)	28, 29
55–64	1.30% (0.43%)	0.83% (0.48%)	28, 29
65–74	3.45% (0.68%)	2.06% (0.49%)	28, 29
75+	20.79% (1.90%)	12.40% (1.05%)	28, 29
Annual smoking‐related health‐care costs after the first year			
Male age group (years)	Continuing smokers	Ex‐smokers	
35–44	£54.48 (£0)	£16.57 (£0)	12, 32, 33
45–54	£54.48 (£0)	£16.57 (£0)	12, 32, 33
55–64	£181.97 (£0)	£64.99 (£0)	12, 32, 33
65–74	£315.75 (£0)	£83.82 (£0)	12, 32, 33
75+	£535.22 (£0)	£105.36 (£0)	12, 32, 33
Female age group (years)	Continuing smokers	Ex‐smokers	
35–44	£41.31 (£0)	£10.72 (£0)	12, 32, 33
45–54	£41.31 (£0)	£10.72 (£0)	12, 32, 33
55–64	£119.83 (£0)	£40.95 (£0)	12, 32, 33
65–74	£249.03 (£0)	£71.25 (£0)	12, 32, 33
75+	£470.69 (£0)	£103.18 (£0)	12, 32, 33
Annual QALY gain after the first year			
Male age group (years)	Continuing smokers	Ex‐smokers	
35–44	0.889 (0.007)	0.908 (0.005)	30
45–54	0.841 (0.007)	0.861 (0.005)	30
55–64	0.780 (0.008)	0.803 (0.005)	30
65–74	0.756 (0.008)	0.781 (0.006)	30
75+	0.710 (0.009)	0.737 (0.006)	30
Female age group (years)	Continuing smokers	Ex‐smokers	
35–44	0.870 (0.007)	0.889 (0.004)	30
45–54	0.830 (0.007)	0.850 (0.005)	30
55–64	0.763 (0.008)	0.784 (0.005)	30
65–74	0.751 (0.008)	0.773 (0.006)	30
75+	0.676 (0.009)	0.700 (0.007)	30

QALY = quality‐adjusted life‐years; SE = standard error.

The model reported a life‐time ICER of a one‐off use of EC, compared with NRT, as smoking cessation aid in English SSS setting from an NHS and PSS perspective. For a probabilistic sensitivity analysis, beta distribution was assigned to parameters for probabilities and gamma distribution to those for costs and QALYs. Monte Carlo simulation was used to randomly draw values for the parameters from their assigned distribution and the expected values of costs and QALYs were calculated. The process was repeated 10 000 times and the results were presented by CEP and CEACs.

All analyses were undertaken using Stata SE version 15.0, with the exception of the Markov model, which was programmed in Microsoft Excel 2016.

## Results

### Treatment cost

The training cost amounted to £4.40 per participant for the EC arm and zero cost for the NRT arm.

Sessions 1 and 2 lasted for 30 minutes each and the following sessions were estimated at 20 minutes. The cost of behavioural support sessions was £80 [standard deviation (SD) = £12] per participant in the EC arm (mean number of sessions: 5.5, SD = 1.0) and £77 (SD = £15) per participant in the NRT arm (mean number of sessions: 5.2, SD = 1.2). Information sheets for the use of EC cost £0.09 per participant and pharmacy lists for redeeming NRT cost £0.05 per participant. LOR was £0.01 each and issued a total of 732 times. Forty‐two participants in the EC arm were given One Kit 2016 at £30.54 per kit. One participant did not accept the kit. Thirty participants requested an extra bottle of e‐liquid costing £1.34 each. The mean cost of products was £20 (SD = £4) per participant in the EC arm, and £124 (SD = £67) per participant in the NRT arm. The delivery cost was therefore £100 (SD = £13) per participant in the EC arm and £201 (SD = £77) per participant in the NRT arm.

### Missing data

In the NRT arm, 59% (265 of 447) participants completed health service use section of 6‐month follow‐up questionnaire, and in the EC arm, 69% (304 of 439) participants did so (Pearson's χ^2^ test *P* = 0.002). This rate at 12‐month follow‐up was 62% (277 of 447) in the NRT arm and 71% (312 of 439) in the EC arm (Pearson's χ^2^ test *P* = 0.004). The missing data pattern showed that most missed the entire section rather than single items (Supporting information, Tables S2 and S3). The cost, expenses and EQ‐5D‐5 L variables at 6‐ and 12‐month follow‐ups all required imputation. The highest level of the missing data was 35% at 6 months (Supporting information, Table S4). The number of imputation was therefore set to 35. Unless otherwise specified, analyses were performed on the 35 imputed data sets.

### Primary analysis

Table 2 (left) summarizes the results of the primary analysis. The mean cost of treatment was £201 (SE = £4) per participant in the NRT arm and £105 (SE = £1) in the EC arm. The mean total costs were £1116 (SE = £163) in the NRT arm and £1174 (SE = £147) in the EC arm during the 12‐month trial period. After adjustment, the mean total costs in the EC arm was £11 [95% confidence interval (CI) = –£104 to £147] higher than in the NRT arm. The mean QALYs in the NRT arm were 0.882 (SE = 0.009) and 0.886 (SE = 0.008) in the EC arm. After adjustment, the mean QALYs in the EC arm were 0.010 (95% CI = –0.003 to 0.023) higher than in the NRT arm. The ICER was calculated at £1100 per QALY gained indicating that, compared with the NRT arm, the EC arm spent an extra £1100 to yield an additional QALY per person. If the decision‐maker is willing to pay £1100 and above for an additional QALY per person, the EC treatment would be considered the cost‐effective option.

**Table 2:** Incremental cost‐effectiveness analysis for the primary analysis (left) and the complete case analysis (right).

	Primary analysis		Complete case analysis	
	NRT (*n* = 447)	EC (*n* = 439)	NRT (*n* = 204)	EC (*n* = 254)
Costs during the trial period	Mean (SE)		Mean (SD)	
Treatment cost	£201 (£4)	£105 (£1)	£216 (£73)	£108 (£10)
Smoking cessation costs	£77 (£13)	£48 (£11)	£71 (£165)	£46 (£190)
Health‐care costs	£839 (£162)	£1022 (£147)	£1051 (£4611)	£1110 (£3018)
Total costs during the trial period	£1116 (£163)	£1174 (£147)	£1339 (£4616)	£1264 (£3031)
Incremental costs, mean (95% CI)				
Adjusted difference in total costs during the trial period	£11 (−£104 to £147)		–£96 (−£304 to £81)	
Quality of life during the trial period	Mean (SE)		Mean (SD)	
QALYs	0.882 (0.009)	0.886 (0.008)	0.893 (0.162)	0.883 (0.170)
Incremental QALYs, mean (95% CI)				
Adjusted difference in QALYs	0.010 (−0.003 to 0.023)		0.003 (−0.018 to 0.023)	
Incremental cost‐effectiveness ratio (ICER), mean (uncertainty)				
ICER at 12 months post‐quit date	£1100 per QALY gained (Fig. 2 upper left Cost‐effectiveness plane)		EC dominant (less costly, more effective) (Fig. 2 lower left, cost‐effectiveness plane)	

QALY = quality‐adjusted life‐years; SD = standard deviation; SE = standard error; CI = confidence interval; NRT = nicotine replacement therapy; EC = e‐cigarette.

**Figure 2:**
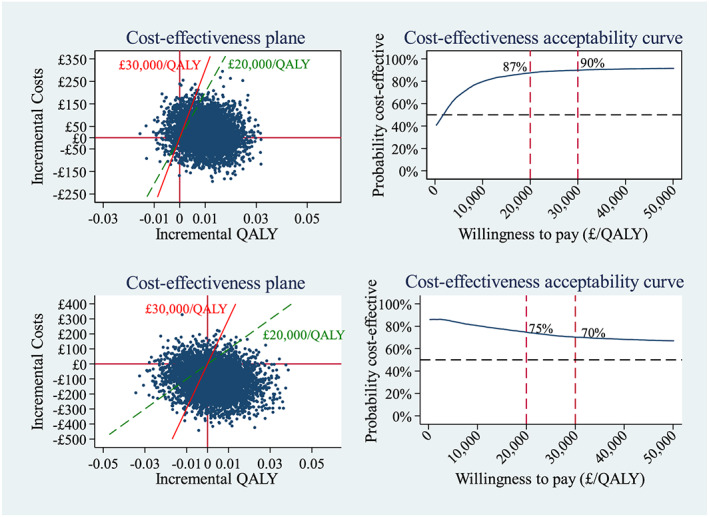
Cost‐effectiveness plane and cost‐effectiveness acceptability curve for the primary analysis (upper) and complete case analysis (lower), QALY=, quality‐adjusted life‐years [Colour figure can be viewed at wileyonlinelibrary.com]

Figure 2 (upper) shows the CEP and CEACs constructed with bootstrapped replicates. The overall majority (93%) fell on the right of the *y*‐axis on the CEP, indicating a highly likely effective intervention, while the existence of difference in costs was less certain. However, most of the replicates fell below the WTP thresholds, suggesting that the EC was likely to be more cost‐effective than the NRT. The CEACs further illustrated this point by estimating the probability of EC being cost‐effective in comparison with NRT to be 87% at £20 000/QALY and 90% at £30 000/QALY.

### Secondary analyses

#### Complete case analysis

Table 2 (right) summarizes the results of the CCA, which was undertaken on 254 of 439 (58%) participants in the EC arm and 204 of 447 (46%) in the NRT arm. The treatment cost was £216 (SD = £73) per participant in the NRT arm and £108 (SD = £10) in the EC arm. After similar adjustment to the primary analysis, the incremental costs became negative, suggesting a cost saving in the EC arm. The adjusted mean difference in QALYs was 0.003 (95% CI = –0.018 to 0.023), with the EC arm slightly higher. The mean ICER indicated a dominance situation, where the EC arm was less costly but more effective. Figure 2 (lower) shows that the difference in QALYs became more uncertain, while the overall majority (86%) fell below zero for the difference in costs. The probability of cost‐effectiveness was 75% at £20 000/QALY and 70% at £30 000/QALY. Table 3 compares the estimated mean costs to the SSS and NHS and mean EQ‐5D‐5L utility in the CCA with the primary analysis. Both arms indicated slightly higher mean costs to the SSS and NHS, but the difference in the NRT arm was more prominent. Mean utility in the CCA appeared consistently higher than in the primary analysis in the NRT arm.

**Table 3:** Comaprison of costs to the NHS and EQ‐5D‐5 L utility values between the imputed data and the complete case.

Analysis	NRT		EC	
	*n*	Mean	*n*	Mean
Costs to the SSS and NHS				
In the 6 months before trial				
Imputed (SE)	447	£645 (£109)	439	£539 (£62)
Complete case (SD)	204	£688 (£2811)	254	£593 (£1490)
In the 12‐month trial period				
Imputed (SE)	447	£915 (£163)	439	£1069 (£147)
Complete case (SD)	204	£1123 (£4621)	254	£1156 (£3032)
EQ‐5D‐5 L utility				
Baseline				
Imputed (SE)	447	0.878 (0.008)	439	0.868 (0.009)
Complete case (SD)	204	0.885 (0.162)	254	0.868 (0.193)
Six months				
Imputed (SE)	447	0.882 (0.011)	439	0.888 (0.010)
Complete case (SD)	204	0.897 (0.198)	254	0.882 (0.199)
Twelve months				
Imputed (SE)	447	0.887 (0.011)	439	0.898 (0.011)
Complete case (SD)	204	0.893 (0.205)	254	0.900 (0.202)

NHS = National Health Service; NRT = nicotine replacement therapy; EQ‐5D‐5 L = 5‐level EuroQol 5‐dimension; SSS = Stop Smoking Service; SE = standard error; SD = standard deviation.

#### Comparison of participants’ expenses on smoking cessation between arms

The mean expenses on smoking cessation aids were £158 (SE = £27) per participant in the NRT arm in the 12 months post‐TQD, including £89 (SE = £26) for NRT, £49 (SE = £6) for EC and £20 (SE = £2) for prescription charge. During the same period, the mean expenses were £168 (SE = £11) in the EC arm, including £12 (SE = £5) for NRT, £152 (SE = £10) for EC and £4 (SE = £2) for prescription charge.

#### Long‐term model

**Figure 3:**
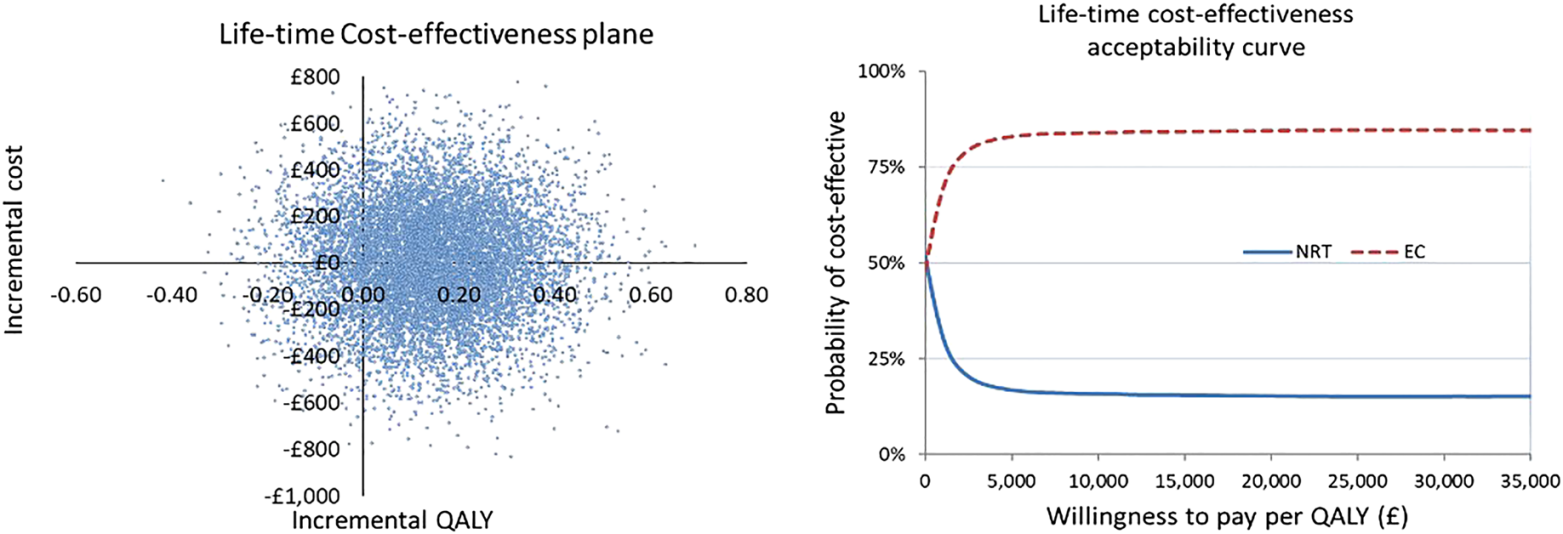
Life‐time cost‐effectiveness plane and cost‐effectiveness acceptability curve. QALY=, quality‐adjuested life‐years [Colour figure can be viewed at wileyonlinelibrary.com]

The cohort of 1000 people entered the model at the age of 41 years. The mean life‐time smoking‐attributable costs were estimated at £3175 (SE = £161) per smoker who used NRT as cessation aid and £3184 (SE = £169) per smoker who used EC. The mean QALYs were estimated at 24.14 (SE = 0.31) per person who used NRT and 24.28 (SE = 0.31) per person who used EC. The ICER was calculated at £65 per QALY gained by using EC as smoking cessation aid, in comparison with NRT. Figure 3 shows the life‐time CEP and CEACs constructed from the probabilistic sensitivity analysis. It indicated that the life‐time costs were likely to be similar between using EC and NRT, but the EC intervention resulted in a positive QALY gain with high certainty. The probability of EC being more cost‐effective than NRT remains at 85% at £20 000 and £30 000 per QALY WTP threshold.

## Discussion

The mean treatment cost was £201 (SE = £4) per participant in the NRT arm and £105 (SE = £1) in the EC arm. The 12‐month ICER in the primary analysis was £1100 per QALY gained from an NHS and PSS perspective, with the probability of cost‐effectiveness being 87% at £20 000 and 90% at £30 000 WTP thresholds. The CCA suggested that the effect of missing data was more prominent in the NRT arm, due possibly to the lower completion rate, which renders the estimates of the NRT arm in the primary analysis less certain. Long‐term modelling estimated a life‐time ICER at £65 per QALY gained, with a probability of 85% that EC is more cost‐effective at both £20 000 and £30 000 per QALY gained. This indicated EC as a highly cost‐effective cessation aid, compared with NRT, as part of the English SSS, from an NHS and PSS perspective. The comparison between participants’ expenses on smoking cessation between arms showed no apparent difference, while the costs of smoking cessation borne by the SSS and NHS were lower in the EC arm. This suggested that the EC intervention could potentially reduce the costs to the SSS and NHS without increasing the financial burden on the smokers’ part.

The sample size of 886 reduced the possibility of random individuals, with particularly high health‐care use being allocated to one arm. However, the 79% 1‐year follow‐up rate, adding to the incomplete rate of health economic section, contributed to a 35% missing data level at the highest. Although multiple imputation and complete case analyses showed consistent conclusions, the high level of missing data makes it less certain as to how cost‐effective the intervention really is, and represents one of the limitations of the study. In addition, the 6‐month recall period for self‐reported health‐care services use and the comprehensive, but long, questionnaire could potentially cause recall bias.

We used the long‐term model to evaluate the EC use in an appropriate time horizon for smoking cessation. It showed a favourable result of EC use due mainly to the significantly higher abstinence rate at 12 months post‐TQD in the EC arm. However, the model did not take into account repeated attempts to quit or the possible long‐term effects of using EC on health and personal finance. There is a lack of evidence on user behaviour regarding EC and the impact of continuous use of EC on health in the long term. While the costs of smoking‐related diseases were better identified and estimated, QALYs were derived from the population tariff based on smoking status, and were not disease‐specific.

Our study provided the initial EC products at no cost to the participants, which is not common practice within SSS at the present time. While the relevant policy change remains uncertain, people who want to quit might ask for advice on the use of EC. This requires staff in the SSS and NHS to be equipped with correct and sufficient information about the potential role of EC in aiding smoking cessation. Decision‐makers should also be aware that the implementation costs of EC treatment was not within the scope of our analysis, but might add an influence if free provision of the EC starter pack is incorporated into standard SSS.

Existing evidence suggests varenicline, as a smoking cessation aid, to be more cost‐effective or even cost‐saving compared with bupropion and NRT 35, 36, 37. While counselling is a cost‐effective treatment for smoking cessation, it has been suggested that counselling plus NRT might be more cost‐effective 38. There are few published RCTs studying EC as a smoking cessation or reduction aid. Hartmann‐Boyce and colleagues identified four 39, 40, none of which compared EC with NRT in the standard SSS settings. To the best of our knowledge, this is the first cost‐effectiveness study comparing EC and NRT as alternative cessation aids as part of the SSS. The relative cost‐effectiveness of varenicline and EC remains unstudied.

The provision of an EC starter pack, compared with using NRT, in a standard SSS for smoking cessation is cost‐effective. There was no evidence in the trial to suggest that the participants’ expenses on smoking cessation aids increased due to the initiation of EC. The long‐term impact on cost‐effectiveness requires further research on the possible health side‐effects of EC.

## Clinical trial registration

ISRCTN60477608.

## Declaration of interests

P.H. has received research grant from and provided consultancy to Pfizer. D.P. has received a research grant from Pfizer. L.D. has provided consultancy for the pharmaceutical industry (2015, 2017) and acted as an expert witness for an e‐cigarette patent infringement case (2015). M.L.G. has received a research grant from Pfizer and served as a member of scientific advisory board to Johnson & Johnson. H.McR. has received honoraria for speaking at smoking cessation meetings and attending advisory board meetings that have been organized by Pfizer and Johnson & Johnson.

## Supporting information


**Table S1** Unit costs of NRT provided in the study, smoking cessation services and pharmacotherapies outside of the study and wider health care services (2015/16 price).
**Table S2** Number of use of smoking cessation help, pharmacotherapies on prescription and other health care services at each follow‐up point, by arm (mean (SD)).
