# Supplementary material for: Cost‐effectiveness of e‐cigarettes compared with nicotine replacement therapy in stop smoking services in England (TEC study): a randomized controlled trial
Source: Addiction. 2019 Dec 4;115(3):507–17. doi: 10.1111/add.14829 (PMC7318206; doi:10.1111/add.14829)
Supplement: Supplementary file 1 — Table S1 Unit costs of NRT provided in the study, smoking cessation services and pharmacotherapies outside of the study and wider health care services (2015/16 price). Table S2 Number of use of smoking cessation help, pharmacotherapies on prescription and other health care services at each follow‐up point, by arm (mean (SD)). [file ADD-115-507-s001.docx]

EC product used in the trial

The original ‘One Kit’: an Aspire device with 2.1 ohm resistance atomiser coil and 650mAh battery, branded by the UK ECIG STORE

E-liquid: 18mg/ml nicotine, ‘Tobacco Royale’, 30ml/bottle

The One Kit 2016: an Innokin device with 1.5 ohm resistance atomiser coil and 1000mAh battery

The use of LORs

There was only one site used LORs to provide NRT. To avoid possible biases, in this site, the participants were given an LOR at the first session and instructed to collect the NRT and bring them to the second session (TQD). On TQD, those randomised to the NRT arm kept their NRT, while those randomised to the EC arm exchanged their NRT for the EC starter kit. In the sites that provided NRT directly, participants received their assigned cessation aid on TQD.

The one site in the trial that used LORs, recruited 296 participants in the NRT arm (586 in total). On the treatment log, there was the records of NRT issued on LORs in this session and from the second session, there was also the records of NRT used since last session by self-report. The matching records for two consecutive sessions where the previous one issued LORs, were available for 250 participants at 1-week post TQD, 105 participants at 2-week post TQD, 98 participants at 3-week post TQD, and 85 participants at 4-week post TQD. Around half of these participants used the same products on LORs (68% at 1-week post TQD, 50% at 2-week TQD, 48% at 3-week post TQD, 61% at 4-week post TQD). The rest either used one of the two products on LORs or did not use any products on LORs.

Among those with matching records for comparison, around half of the participants did not fully comply with the LORs they received after TQD, either failing to redeem or deciding not to use the products after redeeming. However, it is also possible that the participants used more than expected and only recalled the ones that were not on LORs. Given that the costs to the NHS occur at the point of product receipt, the estimated treatment cost for the NRT arm could be overestimated if LORs were often unredeemed. Since LORs were only used in one site and the data were not available for all, the level of this discrepancy should be treated with caution. This discrepancy, if really exists, only affects the cost estimates of NRT arm. It does not imply incompliance to the NRT treatment.

Table S1 Unit costs of NRT provided in the study, smoking cessation services and pharmacotherapies outside of the study and wider health care services (2015/16 price)

| Services | Unit cost | Sources |
| --- | --- | --- |
| NRT products provided in study (NIC + dispensing fee) | | |
| 24hr patch (21mg) | £25/item | [1, 2] |
| 24hr patch (14mg) | £25/item | [1, 2] |
| 24hr patch (7mg) | £25/item | [1, 2] |
| 16hr patch (25mg) | £24/item | [1, 2] |
| 16hr patch (15mg) | £26/item | [1, 2] |
| 16hr patch (10mg) | £26/item | [1, 2] |
| Microtab (2mg) | £17/item | [1, 2] |
| Mouth strip (2.5mg) | £15/item | [1, 2] |
| Gum (4mg) | £15/item | [1, 2] |
| Gum (2mg) | £12/item | [1, 2] |
| Lozenge (4mg) | £15/item | [1, 2] |
| Lozenge (2mg) | £15/item | [1, 2] |
| Nasal spray | £41/item | [1, 2] |
| Minis (4mg) | £17/item | [1, 2] |
| Minis (1.5mg) | £15/item | [1, 2] |
| Inhalator | £33/item | [1, 2] |
| Mouth spray | £26/item | [1, 2] |
| Smoking cessation help | | |
| GP | £36/Session | [3] |
| NHS SSS | £17/Session | [3, 4] |
| NHS Smoking Helpline service | £7/Call | [3, 5] |
| Pharmacotherapies (NIC + dispensing fee) | | |
| Patch | £27/item | [1, 2] |
| Gum | £15/item | [1, 2] |
| Microtab | £19/item | [1, 2] |
| Inhaler | £36/item | [1, 2] |
| Lozenge | £17/item | [1, 2] |
| Spray | £30/item | [1, 2] |
| Mouthstrip | £17/item | [1, 2] |
| Varenicline | £37/item | [1, 2] |
| Bupropion | £40/item | [1, 2] |
| Health care services use | | |
| A & E department | £162/Attendance | [6] |
| Hospital outpatient | £135/Appointment | [6] |
| Hospital inpatient | £606/Night | [6] |
| Day case | £733/Episode | [6] |
| Emergency ambulance | £96/Use | [6] |
| GP (in office) | £31/Consultation | [3] |
| Practice nurse (in office) | £9/Visit | [3, 7] |
| GP (home visit) | £56/Consultation | [3, 7] |
| Practice nurse (home visit) | £17/Visit | [3, 7] |
| Prescription (NIC + dispensing) | £22/prescription | [2] |
| Prescription charge | £8.20/item | [8] |

Table S2: Number of use of smoking cessation help, pharmacotherapies on prescription and other health care services at each follow-up point, by arm (mean (SD))

|  | NRT | | | EC | | |
| --- | --- | --- | --- | --- | --- | --- |
|  | Baseline | 6 months | 12 months | Baseline | 6 months | 12 months |
| Smoking cessation help | n=447 | n=270 | n=285 | n=439 | n=308 | n=314 |
| GP | 0.17 (0.55) | 0.10 (0.45) | 0.18 (1.01) | 0.12 (0.47) | 0.04 (0.35) | 0.04 (0.36) |
| NHS SSS | 0.16 (0.53) | 0.16 (0.74) | 0.47 (1.99) | 0.18 (1.30) | 0.19 (1.62) | 0.26 (1.47) |
| NHS Smoking Helpline service | 0.02 (0.18) | 0.01 (0.11) | 0.00 (0.06) | 0.03 (0.16) | 0.00 (0.06) | - |
| NRT on prescription | n=447 | n=266 | n=282 | n=439 | n=306 | n=315 |
| Patch | 0.20 (1.09) | 0.41 (1.40) | 0.29 (1.55) | 0.18 (1.05) | 0.17 (1.00) | 0.17 (0.91) |
| Gum | 0.04 (0.52) | 0.06 (0.36) | 0.05 (0.64) | 0.07 (0.96) | 0.00 (0.06) | 0.00 (0.06) |
| Microtab | - | - | - | - | 0.00 (0.06) | - |
| Inhalator | 0.30 (3.83) | 0.46 (3.87) | 0.04 (0.49) | 0.24 (3.54) | 0.37 (3.56) | 0.47 (4.96) |
| Lozenge | 0.05 (0.40) | 0.14 (1.13) | 0.05 (0.66) | 0.03 (0.40) | - | 0.05 (0.53) |
| Spray | 0.07 (0.66) | 0.45 (3.00) | 0.21 (2.99) | 0.08 (1.19) | 0.01 (0.08) | - |
| Mouthstrip | - | 0.03 (0.29) | 0.01 (0.12) | - | 0.02 (0.24) | - |
| Varenicline | n=447 | n=271 | n=285 | n=439 | n=306 | n=316 |
|  | 0.12 (0.64) | 0.05 (0.34) | 0.12 (0.67) | 0.06 (0.47) | 0.04 (0.39) | 0.08 (0.52) |
| Bupropion | n=447 | n=271 | n=284 | n=439 | n=306 | n=316 |
|  | 0.00 (0.05) | 0.00 (0.06) | 0.01 (0.13) | 0.00 (0.05) | - | - |
| Health care services | n=447 | n=270 | n=283 | n=439 | n=306 | n=316 |
| A & E | 0.19 (0.52) | 0.11 (0.47) | 0.10 (0.32) | 0.24 (0.80) | 0.11 (0.38) | 0.10 (0.34) |
| Outpatient | 1.04 (5.18) | 0.85 (3.92) | 0.68 (2.55) | 0.73 (2.23) | 0.85 (3.71) | 0.68 (3.07) |
| Inpatient (nights) | 0.30 (2.87) | 0.22 (1.57) | 0.32 (2.80) | 0.23 (1.78) | 0.17 (1.61) | 0.50 (3.58) |
| Day case | 0.16 (0.77) | 0.06 (0.24) | 0.14 (1.45) | 0.13 (0.48) | 0.13 (0.97) | 0.08 (0.35) |
| Emergency ambulance | 0.04 (0.22) | 0.03 (0.38) | 0.04 (0.20) | 0.06 (0.31) | 0.01 (0.08) | 0.03 (0.16) |
| GP (in office) | 2.05 (3.17) | 1.40 (2.98) | 1.37 (2.22) | 2.04 (3.69) | 1.41 (2.58) | 1.52 (2.95) |
| Practice nurse (in office) | 0.56 (1.84) | 0.51 (2.02) | 0.31 (0.69) | 0.72 (2.04) | 0.49 (1.88) | 0.37 (0.87) |
| GP (home visit) | 0.01 (0.08) | - | 0.00 (0.06) | 0.00 (0.05) | 0.02 (0.15) | 0.02 (0.23) |
| Practice nurse (home visit) | 0.01 (0.19) | 0.01 (0.18) | 0.17 (2.85) | - | 0.01 (0.08) | 0.01 (0.11) |
| Prescription | 3.13 (6.39) | 2.74 (5.85) | 2.98 (4.78) | 2.92 (5.55) | 2.55 (6.02) | 2.53 (4.43) |

Table S3: Proportion of five levels of each domain of EQ-5D-5L at each time point, by arm

| Dimensions | | Mobility | | Self-care | | Usual activities | | Pain/ Discomfort | | Anxiety/ Depression | |
| --- | --- | --- | --- | --- | --- | --- | --- | --- | --- | --- | --- |
|  | | NRT | EC | NRT | EC | NRT | EC | NRT | EC | NRT | EC |
| Baseline | | | | | | | | | | | |
| NRT: 447  EC:  439 | 1 | 389 | 369 | 428 | 415 | 388 | 380 | 313 | 302 | 316 | 305 |
|  |  | 87% | 84% | 96% | 95% | 87% | 87% | 70% | 69% | 71% | 69% |
|  | 2 | 23 | 33 | 10 | 12 | 30 | 32 | 68 | 72 | 74 | 82 |
|  |  | 5% | 8% | 2% | 3% | 7% | 7% | 15% | 16% | 17% | 19% |
|  | 3 | 20 | 26 | 6 | 8 | 21 | 17 | 46 | 46 | 44 | 43 |
|  |  | 4% | 6% | 1% | 2% | 5% | 4% | 10% | 10% | 10% | 10% |
|  | 4 | 15 | 11 | 3 | 4 | 8 | 9 | 19 | 13 | 11 | 5 |
|  |  | 3% | 3% | 1% | 1% | 2% | 2% | 4% | 3% | 2% | 1% |
|  | 5 | 0 | 0 | 0 | 0 | 0 | 1 | 1 | 6 | 2 | 4 |
|  |  | 0% | 0% | 0% | 0% | 0% | 0% | 0% | 1% | 0% | 1% |
| 6 months | | | | | | | | | | | |
| NRT: 270  EC:  306 | 1 | 236 | 264 | 251 | 286 | 241 | 274 | 214 | 241 | 203 | 241 |
|  |  | 87% | 86% | 93% | 93% | 89% | 90% | 79% | 79% | 75% | 79% |
|  | 2 | 15 | 13 | 10 | 6 | 9 | 9 | 16 | 22 | 32 | 29 |
|  |  | 6% | 4% | 4% | 2% | 3% | 3% | 6% | 7% | 12% | 9% |
|  | 3 | 12 | 17 | 6 | 10 | 11 | 18 | 31 | 29 | 26 | 22 |
|  |  | 4% | 6% | 2% | 3% | 4% | 6% | 11% | 9% | 10% | 7% |
|  | 4 | 6 | 11 | 1 | 3 | 6 | 2 | 5 | 12 | 5 | 11 |
|  |  | 2% | 4% | 0% | 1% | 2% | 1% | 2% | 4% | 2% | 4% |
|  | 5 | 1 | 1 | 2 | 1 | 3 | 3 | 4 | 2 | 4 | 3 |
|  |  | 0% | 0% | 1% | 0% | 1% | 1% | 1% | 1% | 1% | 1% |
| 12 months | | | | | | | | | | | |
| NRT: 281  EC:  314 | 1 | 245 | 274 | 267 | 297 | 255 | 289 | 226 | 251 | 220 | 264 |
|  |  | 87% | 87% | 95% | 95% | 91% | 92% | 80% | 80% | 78% | 84% |
|  | 2 | 14 | 15 | 5 | 7 | 8 | 6 | 22 | 27 | 30 | 11 |
|  |  | 5% | 5% | 2% | 2% | 3% | 2% | 8% | 9% | 11% | 4% |
|  | 3 | 7 | 10 | 4 | 3 | 6 | 7 | 21 | 22 | 25 | 31 |
|  |  | 2% | 3% | 1% | 1% | 2% | 2% | 7% | 7% | 9% | 10% |
|  | 4 | 13 | 14 | 3 | 5 | 7 | 9 | 8 | 11 | 4 | 6 |
|  |  | 5% | 4% | 1% | 2% | 2% | 3% | 3% | 4% | 1% | 2% |
|  | 5 | 2 | 1 | 1 | 2 | 4 | 3 | 3 | 3 | 2 | 2 |
|  |  | 1% | 0% | 0% | 1% | 1% | 1% | 1% | 1% | 1% | 1% |

Table S4: Number and proportion of missing values of the variables included in the imputation model

| Variables | Number of missing values | Proportion of missing values |
| --- | --- | --- |
| Age | 0 | 0% |
| Gender | 0 | 0% |
| Study site | 0 | 0% |
| Entitlement of free prescriptions | 0 | 0% |
| Ethnicity | 0 | 0% |
| Intervention training cost | 0 | 0% |
| Intervention delivery cost | 0 | 0% |
| Baseline FTCD | 0 | 0% |
| Baseline smoking cessation help cost | 0 | 0% |
| 6 months smoking cessation help cost | 307 | 35% |
| 12 months smoking cessation help cost | 285 | 32% |
| Baseline pharmacotherapies prescription cost | 0 | 0% |
| 6 months pharmacotherapies prescription cost | 313 | 35% |
| 12 months pharmacotherapies prescription cost | 288 | 33% |
| Baseline expenses on NRT over the counter | 0 | 0% |
| 6 months expenses on NRT over the counter | 313 | 35% |
| 12 months expenses on NRT over the counter | 287 | 33% |
| Baseline health care services cost | 0 | 0% |
| 6 months health care services cost | 309 | 35% |
| 12 months health care services cost | 285 | 32% |
| Baseline expenses on e-cigarette | 0 | 0% |
| 6 months expenses on e-cigarette | 309 | 35% |
| 12 months expenses on e-cigarette | 292 | 33% |
| Baseline prescription charges on pharmacotherapies | 0 | 0% |
| 6 months prescription charges on pharmacotherapies | 185 | 21% |
| 12 months prescription charges on pharmacotherapies | 172 | 19% |
| Baseline utility score | 0 | 0% |
| 6 months utility score | 309 | 35% |
| 12 months utility score | 290 | 33% |
| Baseline VAS | 0 | 0% |
| 6 months VAS | 308 | 35% |
| 12 months VAS | 289 | 33% |

1. Prescribing & Medicines Team. Prescription Cost Analysis: England 2016. Health and Social Care Information Centre; 2017 30th March. Available at: https://files.digital.nhs.uk/publicationimport/pub23xxx/pub23631/pres-cost-anal-eng-2016-rep.pdf Archived at: http://www.webcitation.org/766gSvNpi

2. PD1 reports [Internet]. 2017 [cited Oct 10 2017]. Available at: https://www.nhsbsa.nhs.uk/prescription-data/dispensing-data/information-services-pd1-reports Archived at: http://www.webcitation.org/75q4ZZK7q.

3. Curtis L, Burns A. Unit Costs of Health & Social Care 2016. Canterbury: Personal Social Services Research Unit, University of Kent; 2016. Available at: https://www.pssru.ac.uk/pub/uc/uc2016/full.pdf?uc=2016-full Archived at: http://www.webcitation.org/766eDhxAH

4. National Institute for Health and Care Excellence (NICE). Putting NICE guidance into practice - Resource impact report: Stop smoking interventions and services (NG92). 2018 March. Available at: https://www.nice.org.uk/guidance/ng92/resources/resource-impact-report-pdf-4788823645 Archived at: http://www.webcitation.org/766hqbhTb

5. Wu Q, Parrott S, Godfrey C, Gilbert H, Nazareth I, Leurent B, et al. Cost-effectiveness of computer-tailored smoking cessation advice in primary care: a randomized trial (ESCAPE). Nicotine & tobacco research : official journal of the Society for Research on Nicotine and Tobacco. 2014;16(3):270-8.

6. Department of Health. Reference costs 2015-16. 2016. Available at: https://www.gov.uk/government/publications/nhs-reference-costs-2015-to-2016 Archived at: http://www.webcitation.org/766iw6DiA

7. Curtis L, Burns A. Unit Costs of Health and Social Care 2015. Kent: Personal Social Services Research Unit, The University of Kent; 2015. Available at: https://www.pssru.ac.uk/pub/uc/uc2015/full.pdf Archived at: http://www.webcitation.org/6zJ4r87qP

8. Department of Health. Charges for NHS prescriptions, dental charges, elastic stockings and tights, wigs and fabric supports and optical voucher values:Written statement - HLWS346 2015 [cited 2018 Feb 1]. http://www.parliament.uk/business/publications/written-questions-answers-statements/written-statement/Lords/2015-03-11/HLWS346/ Archived at: http://www.webcitation.org/6wu8wxGSR.
